# Exogenously applied ABA alleviates dysplasia of maize (*Zea mays* L.) ear under drought stress by altering photosynthesis and sucrose transport

**DOI:** 10.1080/15592324.2025.2462497

**Published:** 2025-02-05

**Authors:** Zizhu Jiang, Yu Peng Li, Ping Zhuo Gai, Jinsheng Gao, Lijian Xu

**Affiliations:** College of Advanced Agriculture and Ecological Environment, Heilongjiang University, Harbin, China

**Keywords:** Maize, drought stress, abscisic acid, ear, photosynthesis, sucrose transport

## Abstract

Drought stress inhibits the development of maize ears. Abscisic acid (ABA) is a plant hormone that can regulate the physicology metabolism under abiotic stress. In this study, maize varieties Zhengdan 958 (ZD958) and Xianyu 335 (XY335) with different filling stages were used as materials. Three treatments were set in the filling period: normal irrigation (CK), drought stress (stress); exogenous ABA + drought stress (ABA+stress). They were used to study the physiological regulation of exogenous ABA on maize ears development during drought stress. Exogenous ABA inhibited bald tip and the decline of maize plant biomass, and increased the number and weight of grains per ear at harvest under drought stress by regulating photosynthetic pigment content (Chla, Chlb, Car), gas exchange parameters (Pn, Tr, gs, Ci, Ls), Chla fluorescence parameters (Fv/Fm, ФPSII, ETR, qP, NPQ), chloroplast structure and function, photosynthetic enzyme activity, and the transcription level of genes coding SUTs (ZmSUT1, ZmSUT2, ZmSUT4, ZmSUT6). There was a significant correlation between physiological indexes of sucrose loading in maize and yield factors. This study discussed the mechanism of exogenous ABA alleviating maize ear dysplasia at grain filling stage under drought stress from the perspective of photosynthesis and sucrose transport.

## 1. Introduction

Maize is one of the food crops with the highest annual total output in the world.^1^ It is of great significance to ensure food security, maintain regional national economic stability and provide clean energy. The change of accumulated temperature and precipitation pattern in the growing season caused by global warming is the main factor limiting agricultural production, including the improvement of maize yield and quality. In the main corn producing areas, water shortage during the filling period leads to a yield loss.^2^ For rain fed agricultural areas, how to quickly transfer the existing assimilates to the grain during the filling stage to alleviate the yield loss is the most practical drought crisis management strategy. At present, spraying exogenous substances is one of the main methods to reduce this loss, which is based on the comprehensive analysis of carbohydrate transport pathways in the process of grain filling.

The yield decline is the result that the limitation of the synthesis and output of photosynthates at source, and the obstacle of unloading at sink. Sucrose (Suc) produced by autotrophs through fixing carbon dioxide constitutes the carbon skeleton of plants, which is transported and unloaded from source tissue (leaves) through vascular bundle phloem to sink organ (grains), so as to meet the carbon reserve required for grain filling, maintain the level of cell metabolism and osmotic function in stress adaptation.^3,4^ Sucrose and its hydrolyzates (glucose, fructose) may participate in gene transcription of important physiological processes (seed germination, flower induction, fertilization) as signal molecules.^5–7^ Understanding the regulation mechanism of photosynthetic capacity on sucrose synthesis under different water conditions is helpful to improve crop yield and quality.^8^ Leaf stomatal closure leads to the decrease of transpiration and diffusion of CO_2_ to chloroplasts, and carbon assimilation inhibition under drought stress.^9^ Severe drought stress destroys the structure and function of chloroplasts. The ability of leaf photosynthesis mainly depends on chloroplasts. The double-layer membrane structure of chloroplasts can maintain the internal environmental balance. Carbon assimilation takes place in the stroma, which has strong fluidity. Plastid globule is a kind of grana that can store lipids. Light reaction is carried out in the protein complex of thylakoid membrane, which can complete the process of absorption, transmission and transformation. At the same time, thylakoid membrane is also the carrier of chlorophyll.^10^ Chlorophyll (Chl) is an important substance for completing photosynthesis, and plays a decisive role in photosynthetic rate.^11^ Photosystem II (PSII) reaction center participates in photochemical reaction, decomposes water molecules into oxygen, and provides energy for CO_2_ assimilation. However, it is vulnerable to abiotic stress.^12^ Chlorophyll a (Chla) fluorescence parameters provide information about photosynthetic performance of plant leaves under drought stress, which can reflect its efficiency. Drought stress causes cell dehydration, decreased cell volume, increased solute concentration and viscosity, and protein aggregation and denaturation. These factors lead to the decline of photosynthetic enzymes activity, including ribulose-1,5-bisphosphate carboxylase/oxygenase (Rubisco), phosphoenolpyruvate carboxylase (PEPC), pyruvate phosphate dikinase (PPDK), and NAD(P)-malic enzyme (NADP-ME), which is an important reason for the decline of carbon assimilation.^13,14^

Different degrees of water deficit (duration and stress intensity) lead to a rapid decrease of assimilates output, an increase in grain abortion, and even the occurrence of empty culms.^15^ Therefore, understanding the loading, transportation, unloading and storage process of sucrose in plants is the key to improve the efficiency of sucrose utilization. Maize phloem loading is a way of transporting sucrose to heterotrophic tissues through the reverse concentration gradient by sucrose transporter (SUTs) across the membrane.^16^ ZmSUT1 which was first cloned in maize is highly expressed in mature leaves and sink tissues.^17^ Oocyte expression studies support that ZmSUT1 has the ability to move Suc, according to the gradient direction, pH and potential on the membrane.^18^ The phenotypic characteristics of ZmSUT1 deletion mutant show that plant growth delays, reproductive maturity is often not achieved, chlorotic leaves accumulate excessive starch and soluble sugars, and the transport of radioactive labeled Suc is blocked.^19^ Therefore, ZmSUT1 is considered to play a role in loading Suc from mature leaves. In subsequent reports, ZmSUT2^20^ and ZmSUT4^21^ are also identified as sucrose transporter genes that control sucrose metabolism.

Abscisic acid, as stress hormone, plays a mediating role on the physiological response of plants to drought. As a fixed organism, plants carry out signal transmission in vivo after being stimulated by stress, and finally reflect the gene expression in the nucleus. This determines the adaptation or succumb of plants to stressors. The application of exogenous ABA induces the expression of drought stress response genes. One is genes encoding important metabolic proteins, such as membrane transporters; the other is encoding regulatory protein genes to improve plant tolerance.^22^ ABA restricts the expansion of guard cells by inducing stomatal movement under drought stress. ABA may participate in the maintenance of ion homeostasis through the interaction between ABA signal components and ion transporters/channels. Exogenous ABA spraying can regulate enzyme activity, coordinate sucrose synthesis and distribution between source and sink.^23^

Previous few studies have explored the relationship between the regulation of exogenous growth regulators (such as ABA) on photosynthetic physiological metabolism and yield factors under drought stress. The mechanism of exogenous ABA regulating sucrose metabolism and ear development of maize under drought stress is unclear. In this study, the method of pot culture in dry shed was used to control the water conditions of maize at filling stage. The effects of exogenous ABA about the production and loading of assimilates in maize and the relationship with ears development were discussed. Taking effective measures to improve drought tolerance is not only the focus of high-yield cultivation but also the key problem to guide the layout of maize production and establish drought resistance management technology system.

## 2. Materials and methods

### 2.1. Plant materials and treatments

Maize hybrid cultivars ZD958 (grain corn variety) and XY335 (grain corn variety) differing during filling period were selected. ZD958 was from Henan Academy of Agricultural Sciences, XY335 was from Pioneer Seed Company, and ABA was from Meidi chemical company (Beijing, China). During the growth period, all treatments were planted in pots, located in the experimental dry shed at Xiangfang district, Harbin, China. The test plastic bucket has uniform specifications (27.5 cm × 20 cm × 25 cm), with holes at the bottom for drainage. The basic fertility of the soil used in the experiment: total nitrogen (1.43 g.kg^−1^), available potassium (254.55 mg.kg^−1^), available phosphorus (33.93 mg.kg^−1^), organic matter (28.6 g.kg^−1^), alkali hydrolyzable nitrogen (186.17 mg.kg^−1^), pH 6.7. Record each growth period from sowing to harvest. Fertilize, clean weeds, and prevent diseases and pests according to unified standards. The plants with the same growth were selected at the tasseling stage and artificially pollinated. Maize plants were treated differently after pollination: (1) normal irrigation treatment (ck), 75%–80% of the maximum field water capacity was used as the irrigation standard of potted soil; (2) drought stress treatment (stress), 50%–55% of that as the potted soil irrigation standard, the duration was set for 5 days and 10 days respectively; (3) exogenous spraying ABA treatment (ABA+stress), spray ABA solution (0.5 mm) on the leaf surface for 2 consecutive days before drought stress, and other conditions were the same as (2). The control plants were sprayed with water. ABA concentration was screened according to the response of plant morphological indicators in the preparation test. Each treatment was repeated five times.

Irrigation water volume was calculated by soil weighing method. Swp-100 portable soil water potential meter (Nanjing Institute of soil research, Chinese Academy of Sciences) was used to monitor the soil water potential value for 24 hours. Fill water in time to maintain the set water potential. Samples (ear leaves and ears) were collected on the 0^th^, 5^th^ and 10^th^ day of experiment respectively. The middle part of the leaves were taken and the leaf vein was removed. The middle part grains of maize ears were taken. Except for the samples used for fresh measurement, the rest samples were stored at −80°C.

### 2.2. Relative water content of leaves

First, weigh the leaves after sampling, then soak them in distilled water for 24 hours (shading), the last weigh them again. React in the drying box at 80°C until the weight does not change, and then weigh them. RWC (relative water content)% = [(fresh weight-dry weight)/(saturated weight-dry weight)] × 100, the result was taken as the average.

### 2.3. Photosynthetic pigment

The leaves were sampled, ground and extracted with 80% acetone. According to the formula of Lichtenthaler,^24^ the content of Chla = 12.21A_663_-2.81A_646_, Chlb = 20.13A_646_-5.03A_663_, and carotenoids (Car) =(1000A470–3.27Ca- 104Cb)/229,measured absorbance values (A) at different wavelengths.

### 2.4. Gas exchange measurements and Chla fluorescence parameters

Using Li-COR 6400 portable photosynthetic apparatus (Inc., Lincoln, USA) according to the instructions to com plete the measurement of the net photosynthetic rate (Pn), intercellular CO_2_ concentration (Ci), stomatal conductance (gs), transpiration rate (Tr) and stomatal limitation (Ls) of functional leaves of maize. The analyses were performed under an air flow rate of 200 μmol s^−1^, 65% humidity, 350 μmol (photon)·m^−2^·s^−1^ light intensity, and 350 μmol·mol^−1^ ambient CO_2_ concentration. Measure various indicators according to the instrument user manual.

The determination of maximal quantum yield of PSII photochemistry (Fv/Fm), effective quantum yield of PSII photochemistry (ΦPSII), electron transport rate (ETR), photochemical quenching coefficient (qP) and non-photochemical quenching (NPQ) needed to be completed with fluorometer PAM-2500 (Walz, Germany) according to the instructions. Firstly, press the M key, and the displayed value is the F_0_ value. The Fm value is measured after the saturation pulse is turned on, and Fv/Fm= (Fm- F_0_)/Fm. Press the Y or S key to measure the Fm’ value, which is the maximum fluorescence quantum under illumination, and Ft is real-time fluorescence. According to the calculation of ΦPSII= (Fm’- Ft)/Fm’=Δ F/Fm’, qP= (Fm’- Ft)/(Fm’- F_0_), NPQ=(Fm – Fm’)/Fm’.

### 2.5. Chloroplast ultrastructure

Cut a cube with a length of 1 mm at the ear leave of maize, and soak it in H_3_PO_4_ buffer (0.1 M, 2.5% glutaraldehyde, pH 7.2); rinse with H_3_PO_4_ buffer for 15 min and repeat for 4 times; soak in 1% osmium acid for 2 hours; rinse with 0.1 M H_3_PO_4_ buffer for 20 min and repeat for 4 times. The dehydration reaction was carried out for 10 min with ethanol (30%, 50%, 70%, 90%) twice each, acetone twice, 100% acetone and resin were mixed in 2:1 (1 hour), 1:1 and 1:2 for 2 hours respectively, no acetone (overnight); embedding treatment; in the oven for 12 hours (45°C) and 48 hours (60°C) respectively; cut the sample to 70 nm; staining; Finally, use electron microscope to take observation.^25^

### 2.6. The activity of photosynthetic enzyme

Leaves (0.5 g) were treated with Tris-H_2_SO_4_ (100 mm, pH 8.0, mercaptoethanol 7 mm, EDTA 1 mm, glycerol 5%, PVP 1%, MgCl_2_ 10 mm) and centrifuged at 10,000 × g for 15 min. The supernatant was stored at low temperature for enzyme activity determination. The absorbance was measured at 340 nm. The composition of the reaction mixture to test for RuBPcase^26^ activity determination was as follows: 1 mm Tris-HCl (pH 8.0), 0.1 M MgCl_2_, 50 mm ATP, 50 mm DTT, 2 M NADH, 1 mm EDTA; 200 μM NaHCO_3_, 15 U/ml 3-phosphoglycerate kinase, 15 U/ml glyceraldehyde-3-phosphate dehydrogenase; H_2_O_2_ (8 × 10^−4^L) were mixed at 30°C for 5 min; 9 mm RuBP. U was the decrease in absorbance value by 0.01 per minute. The composition of reaction mixture for PEPCase^27^ activity determination was as follows: Tris-H_2_SO_4_ (100 mm, pH 9.2, 1 × 10^−3^ L), MgCl_2_ (10 mm, 1 × 10^−4^ L), NaHCO_3_ (10 mm, 01 × 10-^4^ L), NADH (1 g/L, 3 × 10^−4^L), enzyme solution 5 × 10^−4^L, malate dehydrogenase (50 U/ml, 3 × 10^−4^L), H_2_O_2_ (5 × 10^−4^L) were mixed at 30°C for 10 min, PEP (40 mm, 200 μl). U was the decrease of absorbance value by 0.01 per minute. The composition of the reaction mixture for NADP-ME activity determination was as follows: enzyme solution 0.1 ml, Tris-HCl (50 mm, pH 8.0), MgCl_2_ (1 mm), MnCl_2_ (1 mm), EDTA (1 mm), NADP (0.3 mm), and malic acid (5 mm). The composition of the reaction mixture for PPDK (Hiroshi et al., 2001) activity determination was as follows: enzyme solution 0.1 ml, NADH (0.8 mm), H_2_O 0.3 ml, LDH (6 U/L), Tris-HCl (0.06 M, pH 8.3), Mg_2_SO_4_ (6 mm), DTT (6 mm), NH_4_Cl (6 mm), PEP (1.2 mm), Na_4_P_2_O_4_.10 h_2_O (0.5 mm). The activities of NADP-ME and PPDK were calculated as ΔOD× (6.22 _extinction coefficient_ × Δt× P_soluble protein content_)^−1^×V_enzyme solution_.

### 2.7. Morphological and grain yield

When the water content was 14%, 10 ears with similar shape and weight were selected for each treatment to measure the bald tip length, grain number and 1000-grain weight.

### 2.8. RNA isolation and real-time RT-PCR

Total RNA was isolated from maize using TRIzol reagent (Invitrogen, Carlsbad, CA, USA). Specific primers for each gene were designed from the 3’ ends of the gene sequences by Primer Premier 5.0 (Table 1). The TM value should be between and 55–60°C. The cDNA sample was diluted five times as a template for computer detection. The reagent used for real-time quantitative PCR was 2X SG Fast qPCR Master Mix (B639271, BBI) and the instrument was the LightCycler480 II (Roche Molecular Systems Inc. Pleasanton, CA, USA). The fluorescent quantitative PCR test was completed by Meidi biological company (Beijing, China). 2^−ΔΔCt^ was used to express the relative transcription level. On the 0^th^, 5^th^ and 10^th^ day of drought stress, the middle part of the leaf at the ear position was taken for PCR determination of SUTs related genes. The primer designs of related genes are shown in Table 1.

**Table 1.** Primer sequences for real-time RT-PCR.

Gene	Forward sequence	Reverse sequence
*ZmSUT1*	5’-TCGGCAAGGGCAACATCC-3’	5’-TTGGGCAGCAGGAACACG-3’
*ZmSUT2*	5’-GACACTGACTGGATGGGACG −3’	5’-GAACCAACGCCAAGGACA-3’
*ZmSUT4*	5’-GCCATCTGCGTCTACCTTG-3’	5’-GCGATCCGAGTCCTCCTT-3’
*ZmSUT6*	5’-GCGGTGGTCCTCATCGTAGT-3’	5’-GCAGGTTCTTGAGGCACTTG-3’

### 2.9. Statistical analysis

The mean ± standard deviation of the test data was taken as the research object. Using Excel 2010 and SPSS 22.0 to perform statistical analysis and column chart drawing. Fisher’s least significant difference test at the significance level *p* < 0.05 was used for multiple comparisons.

## 3. Results

### 3.1. Relative water content of leaves and biomass

**Figure 1.**
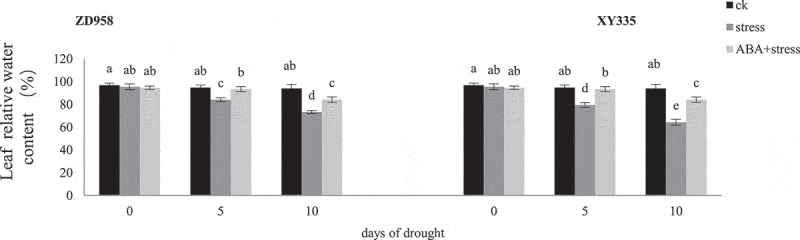
Effects of ABA treatment on the relative water content in leaves of ZD958 and XY335 on the 0^th^, 5^th^, 10^th^ day of drought stress. Every histogram represent the means of five independent measurements, and bars denote the standard error. Different letters indicate significant differences at *p*<0.05.

Compared with ck, the RWC of leaves of ZD958 and XY335 decreased by 11.27%, 22.03% and 16.12%, 31.60% on the 5^th^ and 10^th^ day of drought stress (Figure 1). The decline of leaf relative water content increased with the increase of stress days, and XY335 was more affected by stress. The inhibitory effect of ABA on the decrease of RWC of ZD958 was the most obvious on the 10^th^ day of stress, and XY335 on the 5^th^ day, which was 11.61% and 14.67% higher than that of drought stress alone, respectively.

After 10 days drought treatment, the aboveground dry weight of maize plants decreased significantly (Table 2). The aboveground biomass of XY335 decreased slightly more than that of ZD958, and the decrease of ears dry weight of the two varieties affected by stress was more obvious than that of other parts. Exogenous ABA could effectively alleviate the inhibition of drought stress on maize plant growth, and the effect on XY335 was more obvious, and the mitigation effect on the decline of ear dry weight of the two varieties was stronger than that of other parts. Compared with the control, the leaf area of maize plants decreased for drought stress 10 days, and there was no significant difference between ZD958 and XY335. Exogenous ABA spraying reduced the damage of drought stress on leaf area growth and restored the ability of leaf growth.

**Table 2.** Effects of ABA treatment on plant biomass and leaf area of ZD958 and XY335 on the 10^th^ day of drought stress.

Variety	Treatment	Leaf dry weight (g)	Stem dry weight (g)	Ear dry weight (g)	Leaf area (cm^2^)
ZD958 XY335	ck stress ABA+stress ck stress ABA+stress	93.49±3.21 a 59.26±3.24 c 70.07±3.38 b 90.49±2.73 a 53.32±2.36 c 66.28±2.34 b	55.49±2.50 a 33.26±2.69 c 44.07±4.44 b 54.49±3.78 a 31.32±2.36 c 46.28±2.34 b	75.49±2.50 a 32.46±2.02 c 48.67±2.35 b 74.49±3.68 a 30.72±1.75 c 49.08±2.36 b	5889.09±138.43 a 3472.46±241.06 c 4608.67±135.89 b 5834.49±209.59 a 3392.72±233.95 c 4669.08±193.03 b

Data are expressed as mean ± standard error; a, b and c indicate that the difference is 5% significant

### 3.2. Photosynthetic pigment concentrations

**Figure 2.**
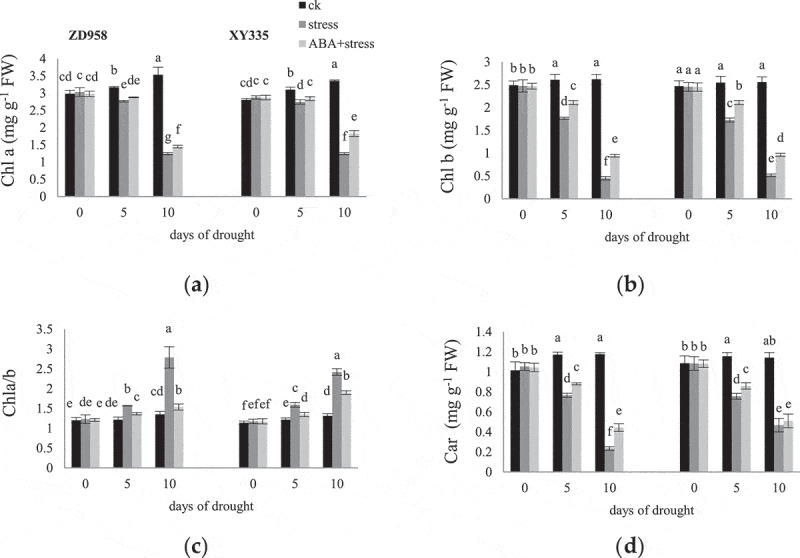
Effects of ABA treatment on Chla (a), Chlb (b), Chla/b (c), car (d) in leaves of ZD958 and XY335 on the 0^th^, 5^th^, 10^th^ day of drought stress. Every histogram represent the means of five independent measurements, and bars denote the standard error. Different letters indicate significant differences at *p*<0.05.

The chlorophyll concentration in ck increased with the grouting process (Figure 2), while in drought stress significantly reduced. Chla/b in ZD958 increased higher than XY335 on the 10^th^ day of stress. Under normal irrigation conditions, the content of Chla and Chlb in ZD958 was higher, while Car content of XY335 was higher. ABA application significantly increased the content of Chla, Chlb and Car of the two varieties under drought conditions. These increases were more significant on the 5^th^ day than the 10^th^ day. ABA significantly reduced Chla/b under drought stress, and its effect on ZD958 was greater than XY335.

### 3.3. Stomatal parameters

**Figure 3.**
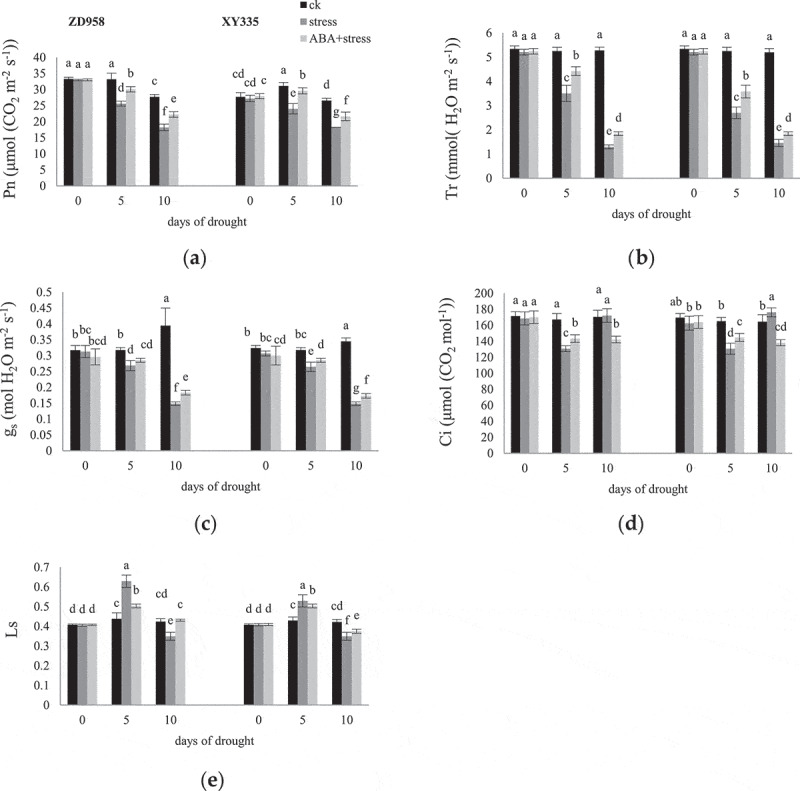
Effects of ABA treatment Pn (a),Tr (b), g_s_ (c), Ci (d), Ls (e) in leaves of ZD958 and XY335 on the 0^th^, 5^th^, 10^th^ day of drought stress. Every histogram represent the means of five independent measurements, and bars denote the standard error. Different letters indicate significant differences at *p*<0.05.

With the continuation of grain filling, the Pn in ck leaves of ZD958 decreased, and that of XY335 first increased and then decreased (Figure 3). The Pn, Tr and gs of the two varieties showed downward trend under drought stress. There were few differences between the cultivars on Tr and gs values, but ZD958 had a higher Pn value than XY335 under both ck and stress treatments. The exogenous application of ABA improved Pn in two cultivars under drought conditions. Furthermore, the effect of ABA elevating Pn was more on the 5^th^ day of stress. Similarly, ABA application also improved g_s_ and Tr values in leaves of the cultivars under stress. The Ci significantly decreased in leaves of ZD958 and XY335 under stress on the 5^th^ day, then increased on the 10^th^ day. ABA improved the Ci on the 5^th^ day, declined the Ci on the 10^th^ day. There were almost opposite changes between Ls and Ci. The effect of ABA on Ls of ZD958 stressed leaves more than that of XY335.

### 3.4. Chlorophyll a fluorescence parameters

**Figure 4.**
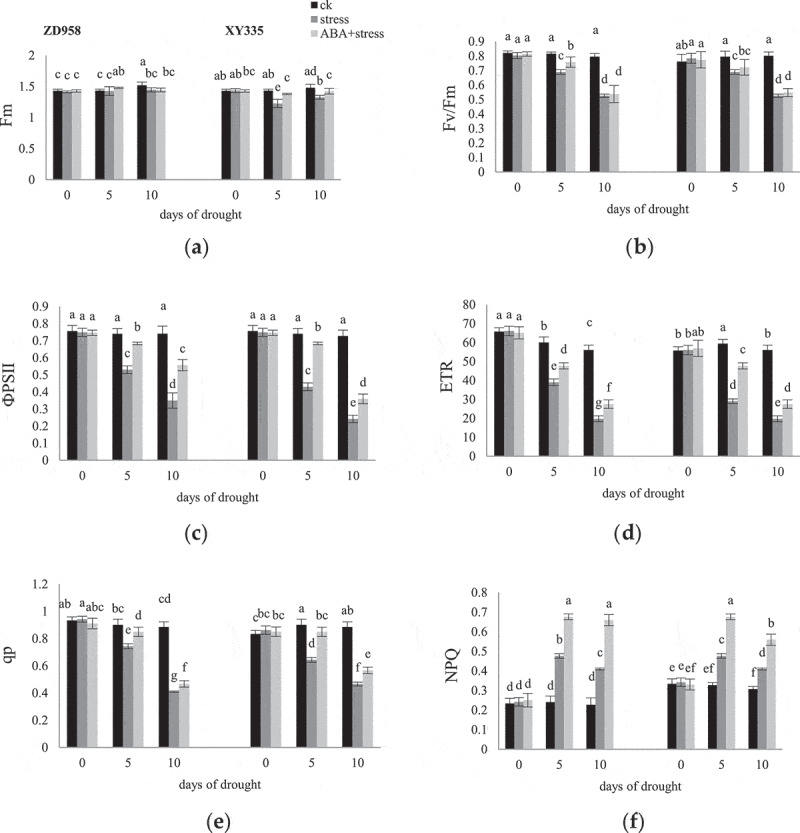
Effects of ABA treatment on Fm (a), Fv/Fm (b), ФPSII (c), ETR (d), qP (e), NPQ (f) in leaves of ZD958 and XY335 on the 0^th^, 5^th^, 10^th^ day of drought stress. Every histogram represent the means of five independent measurements, and bars denote the standard error. Different letters indicate significant differences at *p*<0.05.

With the grouting process (Figure 4), ETR and qP in ZD958 ck group gradually decreased; ETR in XY335 ck group first increased and then decreased, and qP gradually increased. The Fm, Fv/Fm, ФPSII, ETR and qP parameters values were significantly decreased more in both cultivars under drought stress on the 10^th^ day than the 5^th^ day. Under drought stress, the value of NPQ in leaves of the two varieties increased, and the increase in ZD958 was greater than that in XY335. In addition, the application of ABA significantly increased these values, and the impact was greater on the 5^th^ day.

### 3.5. Ultrastructural changes in chloroplasts

**Figure 5.**
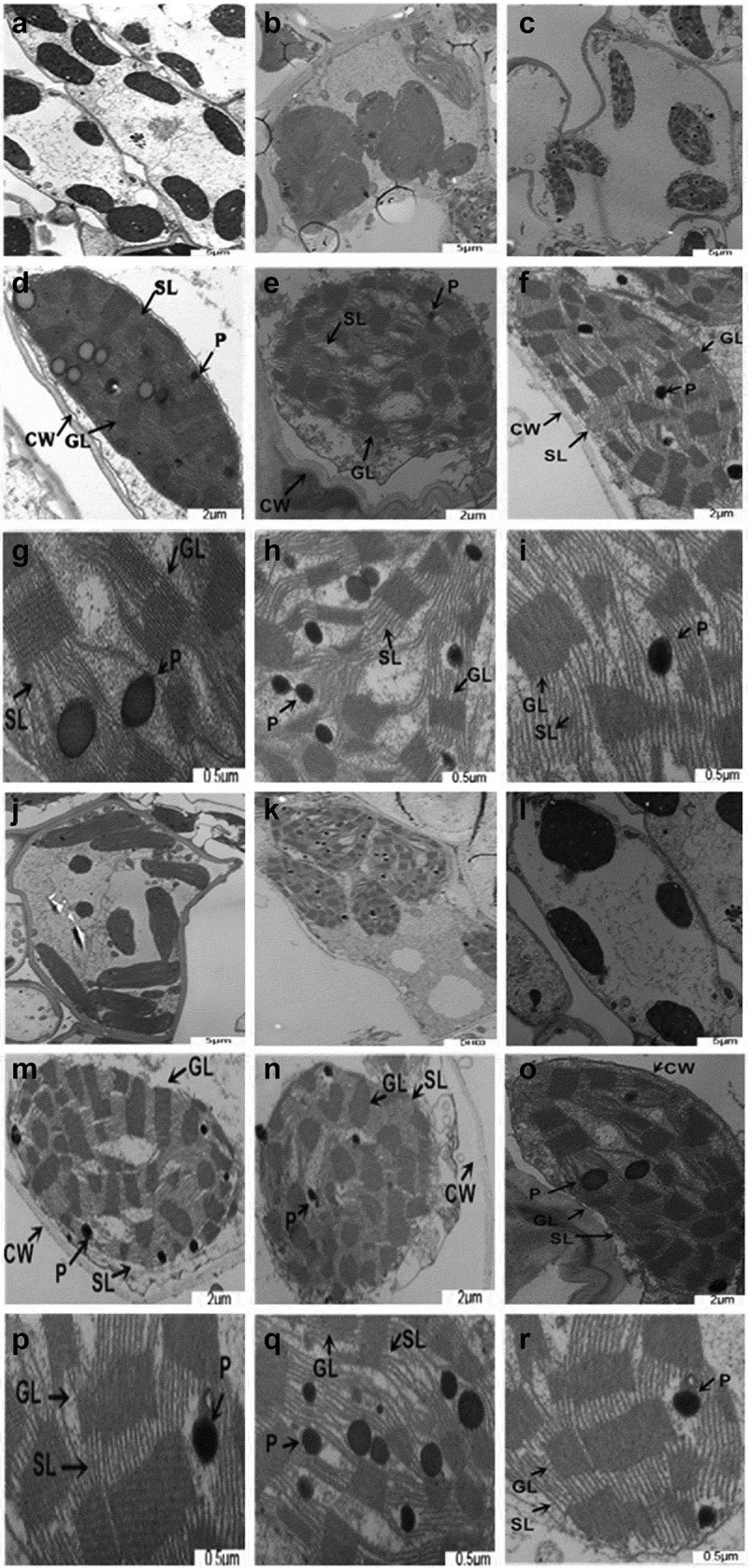
Effects of ABA treatment on ultrastructural changes in chloroplasts in leaves of ZD958 and XY335 on the 10^th^ day of drought stress. A-C, D-F and G-I represent the structural observation of mesophyll cells, chloroplasts and granular thylakoids of ZD958 under ck, stress for 10 days and ABA+stress treatment respectively; J-L, M-O and P-R represent that of XY335.CW stands for cell wall; SL stands for stroma lamellae; GL stands forgrana lamellae; P stands for plastoglobuli. The scale bars for mesophyll cells, chloroplasts and thylakoids are 5, 2, and 0.5μm respectively.

Under normal irrigation conditions, the chloroplast membrane in leaves of ZD958 and XY335 was complete, the shape was uniform oblate, matrix thylakoids and grana were orderly distributed (Figure 5). On the 10^th^ day of stress, chloroplasts expanded and deformed, changed from ellipse to approximate circle, and the chloroplast membrane swelled or disintegrated; plastid globules increased significantly and became larger; stromal thylakoids and grana lamellae were arranged unevenly, with fuzzy morphology, and the number of grana decreased. Compared with drought stress alone, under the action of exogenous ABA, the chloroplasts recovered to a regular oval shape, with a double-layer membrane, and the distribution of grana and thylakoid was more orderly.

### 3.6. The activity of photosynthetic enzyme

**Figure 6.**
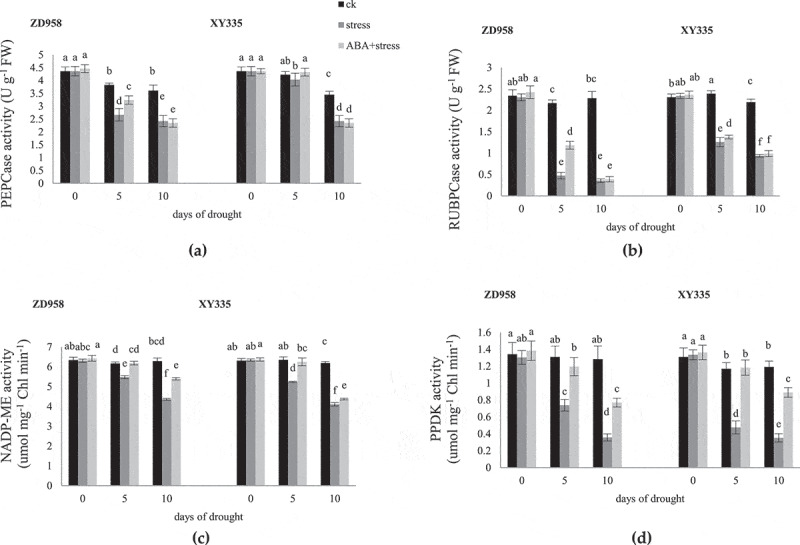
Effects of ABA treatment on the activity of PEPCase (a), RUBPCase (b), NADP-ME (c), PPDK (d) in leaves of ZD958 and XY335 on the 0^th^, 5^th^, 10^th^ day of drought stress. Every histogram represent the means of five independent measurements, and bars denote the standard error. Different letters indicate significant differences at *p*<0.05.

Drought stress significantly reduced the activity of photosynthetic enzyme (Figure 6), and the decline on the 10^th^ day was greater than that on the 5^th^ day. Exogenous ABA increased the enzyme activity of the two varieties, and reached a significant level on the 5^th^ day. Under drought stress, the activity of PEPC and RUBP decreased more than XY335 in ZD958, and PPDK decreased less than XY335. The effect of exogenous ABA on NADP-ME activity in ZD958 was most obvious on the 10^th^ day of drought stress, and the effect on XY335 was most obvious on the 5^th^ day.

### 3.7. The expression of genes encoding SUTs

**Figure 7.**
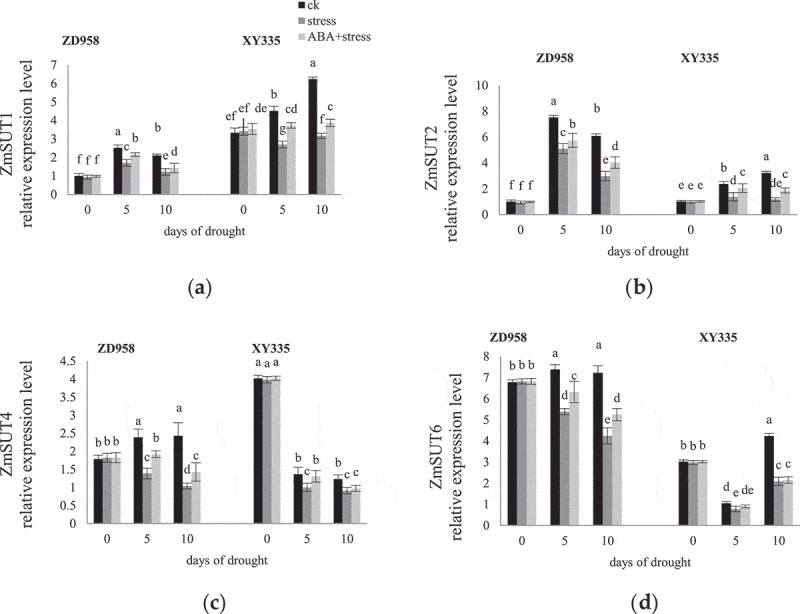
Effects of ABA treatment on the expression levels of ZmSUT1 (a), ZmSUT2 (b), ZmSUT (c), ZmSUT6 (d) encoding SUTs in leaves of ZD958 and XY335 on the 0^th^, 5^th^, 10^th^ day of drought stress. Every histogram represent the means of five independent measurements, and bars denote the standard error. Different letters indicate significant differences at *p*<0.05.

With the process of grouting, the expression levels of ZmSUT1 and ZmSUT2 first increased and then decreased in CK leaves of ZD958, while that of XY335 gradually increased (Figure 7); the expression level of ZmSUT4 and ZmSUT6 of CK leaves in ZD958 increased, while in XY335 decreased or first decreased, then increased. The expression of ZmSUT1 in XY335 was higher than that in ZD958. Drought stress significantly reduced the expression of ZmSUT1, ZmSUT2, ZmSUT4 and ZmSUT6, and the degree of decline on the 10^th^ day was greater than the 5^th^ day. The application of exogenous ABA increased their expression, and the effect on the 5^th^ day was stronger than the 10^th^ day. Exogenous ABA had no significant effect on ZmSUT6 in XY335 under drought stress.

### 3.8. The development of maize ears

**Figure 8.**
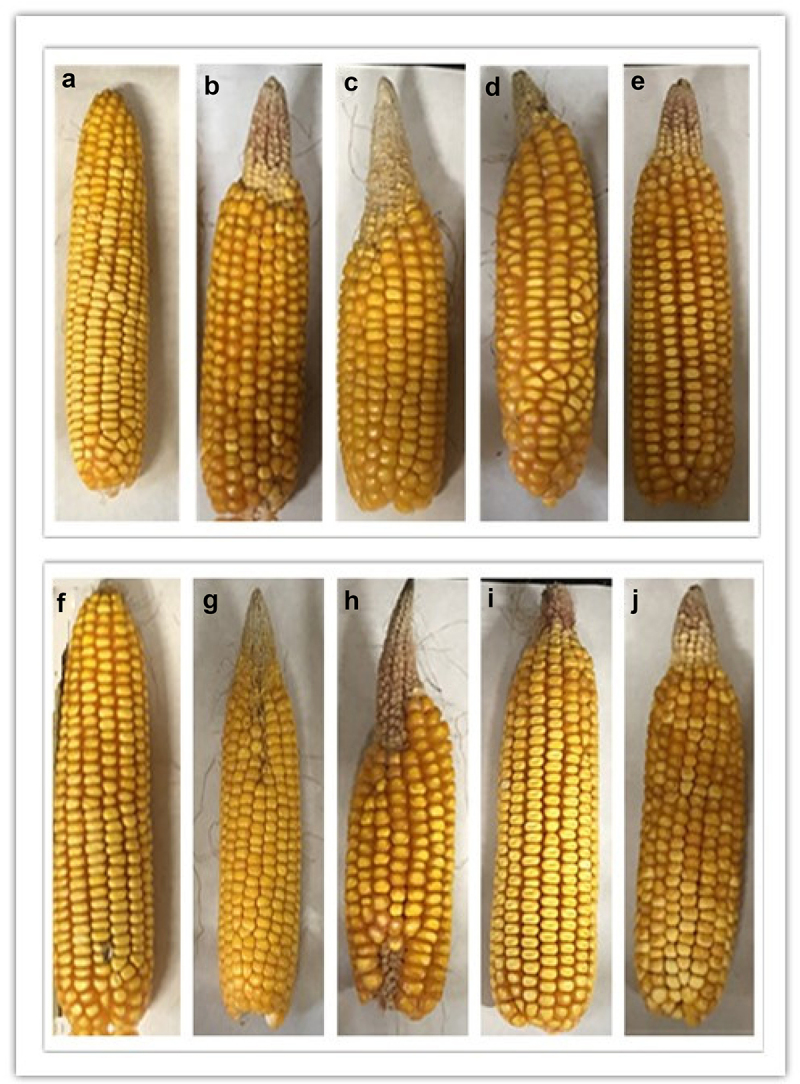
Effects of ABA treatment on the development of maize ears of ZD958 and XY335 on the 0^th^, 5^th^, 10^th^ day of drought stress. A-E stands for ZD958 CK, stress for 5 days, stress for 10 days, ABA+stress for 5 days, ABA+stress for 10 days; F-J stands for XY335 CK, stress for 5 days, stress for 10 days, ABA+stress for 5 days, ABA+stress for 10 days respectively.

Drought stress increased the bald tip length of maize, and the bald tip length increased with the stress days (Figure 8). The bald tip phenomenon was inhibited by ABA, but it could not be completely relieved. The change trend of grain number and 1000-grain weight of two varieties under was similar by stress (Table 3). Drought stress for 10 days had the greatest impact on the decline of yield, and exogenous ABA alleviated it. The correlation analysis between physiological indicators of sucrose transport and yield factors is shown in Table 4.

**Table 3.** Effects of ABA treatment on ear traits and yield components of ZD958 and XY335 for 0, 5, 10 days under drought stress.

Variety	Treatment	Bald tip (cm)	Grain number	1000-grain weight (g)
ZD958 XY335	CK 5 day stress ABA+stress 10 day stress ABA+stress CK 5 day stress ABA+stress 10 day stress ABA+stress	0.2d 4.0b 3.2c 6.0a 4.5b 0.3d 4.5b 3.6c 6.1a 4.8b	700 a 378 c 408 b 266 e 336 d 688 a 336 cd 384 b 250 e 352 c	434.8 a 330.4 c 363.1 b 251.1 e 305.7 d 394.6 a 326.4 c 340.9 b 246.0 e 316.8 cd

**Table 4.** Correlation analysis between maize ear factors and photosynthesis, sucrose transport.

	Bald tip	Grain number	1000-grain weight
Chla	−0.743*	0.655*	0.845**
Chlb	−0.888**	0.813**	0.931**
Chla/b	−0.825**	0.744*	0.898**
Car	−.885**	0.822**	0.903**
Pn	−0.846**	0.765**	0.928**
Tr	−0.944**	0.896**	0.937**
g_s_	−0.867**	0.792**	0.901**
Ci	−0.194	0.300	−0.032
Ls	−0.116	−0.005	0.271
Fm	−0.292	0.295	0.248
Fv/Fm	−0.875**	0.789**	0.907**
ФPSII	−0.887**	0.811**	0.887**
ETR	−0.949**	0.906**	0.955**
qP	−0.840**	0.767**	0.903**
NPQ	0.407	−0.529	−0.261
ZmSUT1	0.084	−0.094	−0.085
ZmSUT2	0.216	−0.310	−0.110
ZmSUT4	−0.782**	0.781*	0.621
ZmSUT6	−0.356	0.349	0.366

*means *p*<0.05, **means *p*<0.01.

Data are expressed as mean ± standard error; a, b and c indicate that the difference is 5% significant.

## 4. Discussion

The impact of drought stress on biomass allocation of damaged plants is easy to attract attention first, but its comprehensive evaluation is still unclear, especially on reproductive organs.^28^ However, it is certain that resource allocation determines the adaptability and reproduction rate of plants in adverse environments, and biomass may be a reliable variable to quantify the allocation of organic matter.^29^ In this study the dry weight of ears decreased more sharply than that of stems and leaves under drought stress during the filling period (Table 2). Similar reports were also reported in the study of reproductive organs of Lycium barbarum L.^30^ The results may be related to ABA mediated photosynthesis of maize plants. In addition, the ear dry weight of the two maize varieties accompanied by an increase significantly with the application of exogenous ABA, and the increase was greater than the stem and leaf dry weight. According to the optimal allocation theory, the resource allocation ratio is controlled by the change of organ relative growth rate and organ size.^31^ Therefore, ABA treatment promoted the flow of photoassimilates to ear of maize, resulting in better ear development related to yield, which was in line with the principle of optimizing plant performance. The maize plants treated with exogenous ABA have a more balanced leaf water status (Figure 1). Because water is an important condition factor to maintain the physiological function and stability of plants, it is inferred that the enhancement of water status caused by the application of ABA is conducive to the growth and development of maize.

Photosynthetic pigments can reflect the tolerance of plants to the environment.^32^ Chla in PSII combines with light collecting complexes CP43 and CP47 for transmitting light energy to reaction center and converting into electric energy.^33^ Chlorophyll b which is only found in photosynthetic antenna complexes is essential for maintaining the stability of the major light-harvesting chlorophyll binding proteins. On the other hand, the excited triplet state of Chl are strong photosensitizers, which will cause the formation of O_2_^·−^ when present in excess. Car exists in the cell membrane, which not only transmits light energy to chlorophyll, but also plays the role of light protection and scavenging reactive oxygen species as a precursor of Signal Transduction under abiotic stress, protecting the membrane from light dependent oxidative damage.^34^ In this study, decreased contents of Chla and Chlb may cause electron transport inhibition, thereby reducing photosynthetic capacity. The increase of Chl a/b ratio indicates that the content of Chlb decreased greater than Chla. This may be due to the conversion of Chlb to Chla during the degradation of Chl, resulting in the increase of Chla content.^35^ Chlorophyll accumulation by ABA application is considered to be a biochemical indicator of stress tolerance, and represents a higher membrane stability.^36^ Previous studies on chlorophyll enzyme and peroxidase have shown that the reduction of chlorophyll under abiotic stress may be due to accelerated decomposition rather than impaired synthesis.^37^ ABA can regulate the expression of NYC1 encoding the reductase which catalyzes the catabolism of Chlb, that is the first part of leaf senescence.^38^ Therefore, exogenous ABA regulates the metabolism of chlorophyll to achieve optimal growth and development, which may be through mediating specific enzyme activities and improving plant antioxidant capacity.

The organic matter produced by photosynthesis maintains the life activities of the whole plant and is very sensitive to water conditions.^39^ The gs and Tr decreased indicating that stomatal opening decreased to prevent water loss.^40^ The change of Pn on the 10^th^ day of drought stress has a more serious impact on the photosynthetic capacity of maize plants than on the 5^th^ day, and may result from stomatal and non-stomatal factors during stress. On the 5^th^ day, gs decreased, Ci decreased, Ls increased, so that stomatal limitation affected Pn decline mainly; on the 10^th^ day, the changes of Ci and Ls were contrary to those recorded on the 5^th^ day, and non-stomata became the main reason. Exogenous ABA can inhibit the decrease of Pn, gs, Tr, and the increase of Ci on the 10^th^ day, and alleviate stomatal and non-stomatal limitations.

Chla fluorescence accounts for 1–2% of the total absorbed light energy, which is proportional to the energy consumed by photosynthetic redox reaction. Based on its statistical electron flow, it can indicate the overall photosynthetic activity, and monitor the process of PSII indirect utilization of excitation energy, which provides valuable evidence for the relevant photosynthetic mechanism.^41^ Chla fluorescence dynamic characteristics of plants is related to yield or plant morphology, and provide drought stress sensitive parameters as the evaluation basis of drought resistance of different genotypes or cultivation measures. They are targeted to resist different stresses, but at present, the corresponding relationship between indicators and stress resistance is not accurately. The ratio of Fv/Fm in plant leaves under normal growth conditions is stable at 0.88.^42^ In this experiment, the significant decrease of Fv/Fm under drought stress indicates that photoinhibition occurs in PSII reaction center, indicating symbiotic stress (drought and photoinhibition) and leaf senescence.^43^ ФPSII or ETR has linear correlation with CO_2_ assimilation quantum yield.^44^ Compared with healthy leaves, early maize leaves under drought stress, the decline of ФPSII is mainly due to the lack of CO_2_ (closed pores) in the leaves, which makes it impossible to effectively use the absorbed radiant energy. ETR calculates the linear transfer of electron flow from H_2_O (PSII) to the Calvin-Benson cycle according to the O_2_ precipitation rate. The decrease of ETR reflects the reduction of photosynthetic electron transfer through PSI and PSII under drought stress, which leads to the insufficient production of ATP and NADPH involved in CO_2_ fixation, and finally limits the CO_2_ reduction process.^45^ Under drought stress, the change of oxygen evolving center (OEC) bound to PSII leads to oxidative damage, and the degradation of reaction center binding protein D1 inactivates PSII reaction center.^46^ ABA can delay leaf senescence, which is related to its participation in D1 protein synthesis and degradation (Wang et al., 2016). Plants protect cell structures from reactive oxygen species by dissipating residual excitation energy.^45^ NPQ reflects the function protection of PSII by measuring the fluorescence heat dissipation.

The Chla fluorescence of the control group of the two maize varieties in this experiment changes differently with the filling process, but the change trend is similar under drought stress. Exogenous ABA act as a signal role to improve Chla fluorescence parameters of maize under drought stress. ABA signal transduction may inhibit the rapid degradation of proteins and mRNAs and stabilize the conversion of proteins by increasing the rate of transcription and translation.^47^ At the same time, ABA has the function of the signal transduction on stress response by control the release of membrane phosphatase inhibitor TLP40 and regulating the phosphorylation and dephosphorylation of PSII protein under drought stress.^48^ Because membrane protein phosphatase activated as the signal of water pressure in chloroplasts is used for the rapid repair process of PSII protein damaged by light. NADP-ME has important catalysis in corn decarboxylation.^49^ The Du test showed that the activity of PPDK decreased by 9.1 times under water stress, which was 2–4 times that of the other enzymes. Therefore, it has been speculated that PPDK is a limiting enzyme for photosynthesis under drought stress.^50^ Exogenous ABA can inhibit the decline of photosynthetic enzyme activity in maize under drought stress, thus reducing yield loss.

Chloroplast is a kind of organelle with double membrane structure. The destruction of its mechanism causes photosynthesis reduction and leaf aging.^51^ Drought stress destroyed the ultrastructure of mesophyll cells related to photochemical activity. Under drought stress, the chloroplast membrane of the leaves of the two varieties of maize was obviously damaged, no longer complete, the grana became larger and indistinguishable, and the matrix lamella was disordered. The increase in the number of plastid globules may be due to thylakoid degradation.^52^ The change of chloroplast structure under drought stress leads to the change of chlorophyll content and proportion, which is believed as the result of lipid peroxidation. The decrease of the ratio of length to width of chloroplasts makes the shape become approximately circular, which also indicates that the function is damaged. Once drought stress breaks the internal balance of plants, inducing premature leaf senescence will limit the increase of yield. Exogenous ABA treatment has a close relationship between chloroplast integrity and photosynthetic performance. Chloroplast is an organelle containing up to 70% leaf protein.^36^ ABA plays a signaling role when plants encounter adversity, changes the expression of chloroplast protein-related genes, and maintains homeostasis by regulating ubiquitin-proteasome system.^53^ Therefore, it is inferred that ABA can alleviate the damage induction of chloroplasts under drought stress through this molecular mechanism.^54^

SUTs, as a functional protein, is responsible for loading, long-distance transportation and unloading of sucrose from the synthetic site to the storage organ, and as a sucrose sensor can adjust the distribution of sucrose between the source and sink according to the environment, ensure plant growth and development and improve stress resistance.^21^ SUTs family genes are divided into different groups, which control the flux of photosynthetic products on the cell membrane in different ways under stress. Studies have found that one of the mechanisms of ABA improving plant stress resistance is to increase the efficiency of sucrose transport by inducing SUTs gene expression, so that carbohydrates accumulate in the sink organs.^55^ ZmSUT1 is located on the plasma membrane of the companion cells, enters the sieve elements through plasmodesmata, and transports sucrose to the distal sink tissue for a long distance.^18^ The expression of ZmSUT1 in leaves of ZD958 and XY335 at the filling stage increased. The accumulation of ZmSUT1 mRNA reflected the transformation from sink to source in new leaves.^53^ The relative expression of ZmSUT1 and ZmSUT4 in XY335 was higher than that in ZD958, indicating that the sucrose transport of XY335 plant was more active and the loading capacity was stronger during the filling period. Under drought stress, the transcription level of ZmSUT1 decreased, which may lead to the yellowing of leaves and excessive accumulation of photosynthetic carbohydrates causing down-regulation of photosynthetic activity.^56^ The growth and development of plants are not coordinated with the availability of nutrient supply, resulting in stunting. Exogenous ABA improved the expression of ZmSUT1 in maize under drought stress, played the dual role of ZmSUT1 loading sucrose in source tissue and recovering any Suc not effectively loaded into companion cells from phloem and xylem, and maintained a low level of extracellular Suc, which may be a mechanism to maintain water flow and turgor pressure in leaves. ZmSUT4 showed high expression induced by low-temperature stress,^21^ which was different from the low expression under drought stress in this experiment. ZmSUT2 is located in the vacuole, which may be responsible for the outflow of sucrose from the vacuole cavity to the cytoplasm, which is consistent with the view that maize uses extracellular pathways to load sucrose phloem.^20^ Previous studies found that the spike length and grain weight of ZmSUT2 mutant decreased.^20^ Therefore, it is speculated that the damage of ZmSUT2 function of maize leaves under drought stress will affect crop yield. The up regulation of ZmSUT2 and ZmSUT4 gene expression by exogenous ABA may reactivate sucrose temporarily stored in vacuoles. However, the effect of ABA on ZmSUT6 is not significant, and the in-depth understanding of its function needs to be further explored.

## 5. Conclusion

ZD958 and XY335 have differences in grouting under normal growth environment, which can be reflected from the value of physiological indicators. Under drought stress, the photosynthetic performance and sucrose transport of the two maize varieties were inhibited to varying degrees, resulting in poor ear development and reduced yield. The application of exogenous ABA improves the transportation efficiency of sucrose between source and sink by improving photosynthesis (sucrose production), up regulating the genes encoding sucrose transporter (sucrose loading) and sucrose INVs (sucrose unloading), so as to alleviate the bald tip of maize and improve the number and weight of grains per ear (Table 4).

## Data Availability

The data presented in this study can be found in the article.
